# Cooperation by ant queens during colony-founding perpetuates alternative forms of social organization

**DOI:** 10.1007/s00265-021-03105-1

**Published:** 2021-11-30

**Authors:** Pierre Blacher, Ornela De Gasperin, Michel Chapuisat

**Affiliations:** Department of Ecology and Evolution, University of Lausanne, Lausanne, Switzerland

**Keywords:** Sociality, Cooperation, Insects, Queen number, Supergene

## Abstract

**Abstract:**

Key social traits, like queen number in eusocial insect colonies, have long been considered plastic, but the recent finding that colony social organization is under strict genetic control in multiple ant lineages challenges this view. This begs the question of which hardwired behavioral mechanism(s) generate alternative forms of social organization during colony development. We addressed this question in the Alpine silver ant, *Formica selysi*, a species with two social forms determined by a supergene. Queens that carry exclusively the *M* haplotype are produced by and live in monogyne (= single-queen) colonies, whereas queens that carry at least one copy of the *P* haplotype are produced by and live in polygyne (= multiple-queen) colonies. With extensive field samplings and laboratory experiments, we show that both types of queens successfully establish colonies independently, without being accompanied by workers, but that they do so in contrasting ways. Monogyne queens were generally intolerant of other queens and founded colonies solitarily, whereas polygyne queens were mutually attracted to each other and mainly founded colonies cooperatively. These associations persisted for months after worker emergence, suggesting that cooperative colony-founding leads to permanent multiple queening. Overall, our study shows that queens of each social form found colonies independently in the field but that *P*-carrying queens are more likely to cooperate, thereby contributing to perpetuate alternative forms of social organization.

**Significance statement:**

Understanding the genetic and behavioral underpinnings of social organization is a major goal in evolutionary biology. Recent studies have shown that colony social organization is controlled by supergenes in multiple ant lineages. But the behavioral processes linking the genotype of a queen to the type of colony she will form remain largely unknown. Here, we show that in Alpine silver ants, alternative supergene genotypes are associated with different levels of social attraction and tolerance in young queens. These hardwired differences in social traits make queens carrying the *P* supergene haplotype more prone to cooperate and form durable associations during independent colony-founding. These findings help explain how genetic variants induce alternative forms of social organization during the ontogeny of a colony. They also illustrate how simple phenotypic differences at the individual level can result in large differences at higher levels of organization.

**Supplementary Information:**

The online version contains supplementary material available at 10.1007/s00265-021-03105-1.

## Introduction

Understanding the genetic and behavioral underpinnings of social organization is a major goal in evolutionary biology (see Schradin 2013). In social insects, as in other group-living animals, social organization has long been considered a phenotypically plastic trait (Stacey and Bock 1978; Koenig et al. 1992; Wcislo 1997; Heinze 2008; Gadau et al. 2009; Maher and Burger 2011; Schradin et al. 2012). This is because key social traits, like mode of colony-founding, levels of cooperation, and number of reproductive queens within colonies, vary with colony development and/or with ecological variables in many species (Ross and Keller 1995; Wcislo 1997; Herbers and Banschbach 1999; Cronin 2001; Ingram 2002; Heinze 2008; Field et al. 2010; Trettin et al. 2014; Cronin et al. 2020). Specifically in ants, colonies founded by a single queen can adopt additional queens as they grow (Bourke and Franks 1995), and colonies founded by multiple queens often retain only one queen after the first cohort of workers emerges (Bernasconi and Strassmann 1999). Moreover, ecological factors, such as nest-site limitation and habitat saturation, are sometimes associated with higher queen number within and across ant populations (Herbers 1986; Ross et al. 1996; Pedersen and Boomsma 1999). Yet queen number has recently been found to be under strict genetic control in several ant lineages (Wang et al. 2013; Purcell et al. 2014; Braim 2015; Brelsford et al. 2020; Yan et al. 2020). In these clades, genetic polymorphisms at large supergenes (i.e., clusters of linked genes) determine whether each colony has one reproducing queen (= monogyne colony) or multiple reproducing queens (= polygyne colony). This strong genetic control of colony queen number raises novel questions on the proximate mechanisms generating alternative forms of social organization. Specifically, by which hardwired behavioral processes do ant queens belonging to each social form perpetuate the monogyne or polygyne colony social organization, respectively?

Across ant species, queens from alternative social organizations typically differ in dispersal and mode of colony-founding (Keller 1991, 1993; Ross and Carpenter 1991; Bourke and Franks 1995; Ross and Keller 1995). Queens from monogyne species usually establish new colonies through independent colony foundation, i.e., without any help from nestmate workers (see Cronin et al. 2013). They disperse on the wing and start colonies either alone (= haplometrosis or solitary founding) or in transient association with other queens (= pleometrosis or cooperative colony-founding; Bernasconi and Strassmann 1999). In contrast, queens from polygyne species often mate in or near their maternal nest into which they are re-adopted. Alternatively, these queens can establish new colonies through dependent colony foundation, i.e., with nestmate workers and, potentially, other queens, after dispersing by walking (Cronin et al. 2013). As a result, queens from polygyne species require low energy reserves and are thus on average smaller, with lower fat reserves, than queens from monogyne species (Keller and Passera 1989; Keller 1991; Stille 1996). It is thus commonly assumed that queens from polygyne species have lost the ability to found new colonies independently (Keller 1993; Keller 1995; but see Hamidi et al. 2017). Yet, in socially polymorphic species, queens from polygyne colonies frequently disperse on the wing (DeHeer et al. 1999; Goodisman et al. 2000; Fontcuberta et al. 2021) and can initiate new colonies independently, at least in the laboratory (DeHeer et al. 1999; DeHeer 2002; Reber et al. 2010; De Gasperin et al. 2020). If both types of queens found nests independently, when and how do the alternative forms of social organization arise during colony development?

Here, we investigated how alternative social forms develop during the early stages of colony establishment in a socially polymorphic ant species, the Alpine silver ant, *F. selysi*. Monogyne and polygyne colonies coexist within populations (Chapuisat et al. 2004). These two colony types differ markedly: monogyne colonies have on average a workforce of 3,000 workers and a lifespan of 10 years, whereas polygyne colonies have on average 30,000 workers and a lifespan of 30 years (Rosset and Chapuisat 2007). Colony social organization is determined by a large and ancient supergene (Purcell et al. 2014; Avril et al. 2019a; Brelsford et al. 2020). This supergene has two haplotypes, *M* and *P* (previously named Sm and Sp). All females in mature monogyne colonies carry only the *M* supergene haplotype, being *MM* homozygous*.* In contrast, females in mature polygyne colonies carry at least one *P* haplotype, being either homozygous *PP* or heterozygous *MP* (Purcell et al. 2014; Avril et al. 2019a). Therefore, *MM* queens (hereafter monogyne queens) are produced by and live in monogyne colonies, whereas *PP* and *MP* queens (hereafter polygyne queens) are produced by and live in polygyne colonies.

The supergene genotype determines colony social organization, but the ontogenetic routes and behavioral processes linking queen genotypes to alternative colony phenotypes remain unknown. While some of the young polygyne queens mate locally and stay in their natal colony (Avril et al. 2019a), others disperse on the wing and mate in swarms, like monogyne queens (Chapuisat et al. 2004; Rosset and Chapuisat 2007; Fontcuberta et al. 2021). These queens are unlikely to be adopted by unrelated colonies (De Gasperin et al. 2021), and laboratory experiments have shown that polygyne queens are able to found colonies independently, albeit with less success than monogyne queens (Reber et al. 2010; Avril et al. 2019b; De Gasperin et al. 2020). Taken together, these results suggest that polygyne queens found colonies independently in the field, but this has not yet been documented. Moreover, we do not know whether they associate with other queens when doing so, nor whether such queen associations are durable or break up after worker emergence (both outcomes occur in ants and vary across species, e.g., Hölldobler and Carlin 1985; Mintzer and Vinson 1985; Mintzer 1987; Trunzer et al. 1998; Bernasconi and Strassmann 1999; Kolmer et al. 2002; Johnson 2004; d’Ettorre et al. 2005; Hölldobler et al. 2011; Helms and Helms Cahan 2012; Eriksson et al. 2019; Lenancker et al. 2019). If polygyne queens form long-lasting associations when establishing colonies, this would provide a mechanism by which these queens perpetuate their colony social organization.

We combined extensive field sampling of incipient colonies with controlled colony-founding experiments in the laboratory to examine (i) whether monogyne and polygyne queens establish colonies independently in the wild, (ii) whether they differ in their propensity to do so solitarily or cooperatively, and (iii) if associations of polygyne queens during colony-founding lead to permanent multi-queened colonies. Overall, these experiments will reveal if the genotype of queens affects their colony-founding behavior in a way that tends to perpetuate alternative forms of social organization.

## Materials and methods

### Independent colony-founding in the field

To assess whether *MM* monogyne and *P*-carrying polygyne queens start nests independently (i.e., without the help of workers) in the field and whether they do so solitary or cooperatively, we collected incipient nests in two populations (Derborence, 46.2806° N, 7.2157° E, 1450 m, and Finges, 46.3138° N, 7.6012° E, 400 m), in Valais, Switzerland, in 2018, 2019, and 2020. We lifted rocks and cautiously dug the small holes found underneath with tweezers. Any small colony found was transferred into a plastic box (15 × 13 cm and 6 cm high). Once colony transfer was complete, we dug deeper in and around the nest cavity, to ensure we had collected the entire colony and that it was not part of a mature colony. We counted the number of queens, workers, larvae, and pupae in each colony and dissected the spermatheca of all queens under a Leica EZ4 stereomicroscope to extract the sperm. To determine the supergene genotype and infer the social origin of each individual, we used a SNP genotyping qPCR assay (for methods, see Fontcuberta et al. 2021). We extracted DNA from queens and brood using Chelex® and from sperm using Qiagen© columns.

To confirm that the incipient colonies had been founded without the help of adult workers from nearby mature colonies, we compared the body size of adult workers found in incipient nests to the one of adult workers born in mature colonies. Workers produced by queens that found colonies independently are usually very small (Hölldobler and Wilson 1990). Workers from 34 mature colonies surrounding incipient colonies were collected in Derborence in 2019. We measured workers under a Leica EZ4 stereomicroscope, using the LAS EZ v2.0 software measuring tool. We recorded the minimum distance between eyes, a good index of total body size (Fortelius et al. 1987).

### Independent colony-founding in the laboratory

#### Sample collection

We sampled callow queens, callow males, and adult workers from previously identified monogyne and polygyne colonies (*n* = 42 and 34, respectively) in Derborence, in July 2017 and 2018. Each colony fragment was transferred into a plastic box (15 × 13 cm and 6 cm high; males and females from the same colony were housed in separate boxes) and kept in 12:12 light:dark cycle, at 25 °C and 60% relative humidity, with food (egg and apple jelly) and water ad libitum.

#### Experiment

We allowed newly mated monogyne and polygyne queens (M and P queens, respectively) to start a colony solitarily or cooperatively with non-nestmate queens. Queens were paint-marked before mating, to allow for subsequent identification. Mating took place outside, during sunny mornings, within plastic containers (35 × 22 cm and 15 cm high) lined with Fluon and covered with a net (see De Gasperin et al. 2020). Each mated queen was housed within a glass tube (length = 16 cm; diameter = 5 mm; ^1^/_3_ filled with water, lined with cotton wool) for a few hours until the start of the experiment. Experimental assays consisted of a pair (two-queen assay) or a triad (three-queen assay) of non-nestmate queens. We used all possible combinations of M and P queens. For two-queen assays, this yielded three treatments: M-M (*n* = 33 assays), M-P (*n* = 31 assays), and P-P (*n* = 30 assays). For three-queen assays, we had four treatments: M-M-M (*n* = 31 assays), M-M-P (*n* = 30 assays), P-P-M (*n* = 30 assays), and P-P-P (*n* = 31 assays). All queens within an assay had mated within the same day, and queen assignment to assays was random regarding the social origin of their mate.

Each pair or triad of queens was placed in an experimental plastic box (15 × 13 cm and 6 cm high), closed by a lid and ¾ filled with humid sand (Spielsand, Colibri®). Neither food nor water was given during the first 2 weeks, to simulate claustral colony-founding conditions, in which queens rely on their body reserves. After this period, jellified water and a small amount of food were placed on a petri dish on the sand surface and were renewed every week. Experimental boxes were maintained at 25 °C, 60% relative humidity, under a 12:12 light:dark regime. We performed behavioral scans twice a day during the first 3 months and then twice a week during 2 more months. In each scan, we recorded (i) whether each queen was alive, (ii) their spatial location (on sand surface or within an excavated nest), and (iii) their spatial proximity to other queens (queens were considered in close proximity when they stand within 2 cm from each other). We recorded the number of workers produced on the last observation day (140 days after the start of the assay). The observations were done blindly with respect to the social origin of the queens.

Queens were categorized as founding queens when they were seen at least once within an excavated nest (i.e., small cavity excavated in the sand, generally located at the bottom of the box and connected to the sand surface via a tunnel). Queens that remained on the sand surface throughout the experiment (or until their death) were categorized as non-founding queens. Founding queens were categorized as co-founding queens if, in one or more scans, they were observed cohabiting peacefully with one or two other queens within the same nest or as solitary founding queens if they nested alone throughout the experiment (or until their death). Once they had settled in a nest, most solitary founding and co-founding queens never exited it (64.4% and 61.1%, respectively) or exited it very occasionally (queens seen on sand surface 3 times or less, 22.8% for both), as expected from claustral queens (Hölldobler and Wilson 1990). None of the co-founding queens that exited the communal nest had founded a new nest solitarily.

## Statistical analysis

Data analyses were performed using generalized linear models (GLMs) and generalized linear mixed models (GLMMs), with Gaussian, Poisson, and binomial error distributions for continuous, count, and binary data, respectively. Data was analyzed using R v4.0.5 (R Core Team 2021) with the package glmmTMB (Brooks et al. 2017), unless otherwise stated. Models’ regression assumptions were assessed with the package DHARMa (Hartig 2020). We used type III sums of squares (SS) for models with interactions and type II SS for models without interactions. Non-significant interaction terms were removed, and the models were then re-calculated. Post hoc tests were corrected for multiple comparisons using the FDR method (Benjamini and Hochberg 1995). Adjusted *p* values are denoted *p*’.

### Independent colony-founding in the field

We compared the body size of workers from incipient and mature colonies with a GLMM (model 1). We included the colony of origin of the workers as a random factor. We compared the brood size (number of larvae, pupae, and workers) between single-queen and multiple-queen colonies with a GLMM (model 2). We included an observation level random effect (olre) to account for overdispersion. We tested with a GLMM if queens and males had mated assortatively with respect to social form (0 = disassortative mating, 1 = assortative mating; model 3). We included the social origin of the queens as a fixed factor and their colony of origin as a random factor.

### Independent colony-founding in the laboratory

#### Are polygyne queens more prone to start colonies cooperatively?

We compared the colony-founding behavior of monogyne and polygyne queens. First, with GLMs, we compared between treatments (i) the proportion of assays in which queens started colonies (0 = no queen founded, 1 = one or several queens founded; a queen was categorized as a founding queen if she was seen within an excavated nest; model 4) and (ii) the proportion of assays with solitary or cooperative colony-founding (0 = solitary founding, 1 = cooperative founding; model 5). We included the treatment as the explanatory variable (three levels in two-queen assays, M-M, M-P, and P-P; four levels in three-queen assays, M-M-M, M-M-P, P-P-M, and P-P-P). In three-queen assays, we considered the majority behavior of queens as the outcome of the assay. The three assays (one M-M-P and two P-P-M) in which both solitary and cooperative colony-founding occurred were thus categorized as cooperative colony-founding.

With GLMMs, we then compared between queens (i) the probability that they started a nest (1 = the queen started a nest; model 6), (ii) the probability that they founded cooperatively (0 = solitarily, 1 = cooperatively; model 7), (iii) the probability that they were spatially close to another queen at the first observation (0 = the queen was alone, 1 = the queen was close to at least one queen; model 8), and (iv) the probability that they died during the first week (1 = the queen was dead; model 9). We included as fixed factors the social origin of the queen (two levels, monogyne or polygyne), the social origin of the other queens in the assay (three levels, monogyne, polygyne, or both), the interaction between these two factors, and the number of queens in the assay (two levels, 2 or 3 queens). We also included the colony of origin of the queen and the assay id nested within the year as random factors. In model 7, we encountered quasi-complete separation (Heinze and Schemper 2002), as characterized by poor model fit, large parameter estimates, and very large group variance. We overcame this problem by fitting the model with the blmer function of the blme package (Chung et al. 2013) and by adding a weak prior on the fixed effects (Quiñones and Wcislo 2015).

#### Is cooperative colony-founding beneficial to queens?

We compared the colony-founding success of the queens with GLMMs. We first assessed the probability that the queens successfully established their colony (model 10), considering that a queen failed when she died or when no brood was produced in her colony before the end of the experiment (140 days from the start of the assay). We then analyzed the number of offspring produced by the queens that succeeded at establishing a colony (model 11). We used the number of workers in the colony at the end of the experiment (= colony size) as the response variable. In all models, we included as fixed factors the social origin of the queen (two levels, monogyne or polygyne), the number of queens with whom the queen had co-founded the colony (three levels, 0, 1, or 2), and the interaction between these factors. As random factors, we included the colony of origin of the queen and her nest id nested within the assay id nested within the year.

#### Does cooperative colony-founding by polygyne queens lead to permanent multi-queened colonies?

We first assessed with a GLMM whether the survival of the queens that founded cooperatively varied according to the social origin of the queens with whom they co-founded (model 12). The survival of each co-founding queen until the end of the experiment was set as the response variable (1 = the queen was dead), and their social origin (two levels, monogyne or polygyne), the social origin of the queen(s) that co-founded with them (two levels, monogyne or polygyne), the interaction between these two factors, and the number of queens that co-founded with them (two levels, 1 or 2) were included as fixed factors. We included the colony of origin of the queen and her nest id nested within the assay id nested within the year as random factors. We did not include queens that co-founded with both monogyne and polygyne queens due to the low sample size for this comparison (*n* = 10). After finding that queens had higher chances of dying when they co-founded with monogyne queens, we tested whether colonies co-founded by at least one monogyne queen had greater likelihood than colonies co-founded by polygyne queens exclusively to become single-queened before the end of the experiment than to remain multi-queened (model 13). For this, we ran a GLM (0 = the colony remained multi-queened, 1 = the colony became single-queened), and we included as explanatory variables the presence of monogyne queen(s) within the colony (two levels, no monogyne queen, at least one monogyne queen), whether colonies produced a brood (two levels, no or yes), and the number of co-foundresses (two levels, 2 or 3). We finally tested with a GLM whether co-founded colonies with and without monogyne queens differed in their likelihood to have a brood and all their co-founding queens alive at the end of the experiment (1 = the colony had a brood and all co-founding queens were alive; model 14). We controlled for the number of queens within colonies by including the number of co-founding queens as a fixed effect in the model.

## Results

### Independent colony-founding in the field

#### Do monogyne and polygyne queens found colonies independently, either solitarily or cooperatively?

Both monogyne and polygyne queens founded incipient nests in the wild (Table 1). Most queens originated from monogyne colonies (91%), in line with the higher proportion of monogyne queens in mating swarms (89.6%; Fontcuberta et al. 2021). All colonies were small, with 2.5 brood on average [min 0, max 11]. Adult workers from incipient colonies were significantly smaller than the ones from surrounding mature colonies (model 1, estimate ± SE = 0.16 ± 0.014, *p* < 0.0001; Online Resource Fig. S1 and Table S1). Specifically, 91.5% of the adult workers from incipient colonies were smaller than the smallest adult worker found in mature colonies. Small colony size and tiny worker body size indicate that the incipient colonies collected in the field had been established independently by queens, with no help from workers of mature colonies.

**Table 1:** Number of incipient nests and queens collected in the field and supergene genotype of each queen and its male mate. One queen that could not be genotyped was excluded from the table

Queen social origin (genotype)	*Monogyne* (*MM)*			*Polygyne (MP)*		
Male genotype	*M*	*P*	NA	*M*	*P*	NA
1-queen nest (*n* = 51)	36	12	2	-	1	-
2-queen nest (*n* = 4)	5	2	1	-	-	-
3-queen nest (*n* = 1)	3	-	-	-	-	-
5-queen nest (*n* = 1)	-	-	-	1	3	1

Both solitary and cooperative colony-founding occurred in the field (Table 1). Among 58 incipient nests, 52 (90%) had 1 queen and 6 (10%) had between 2 and 5 queens, with a median of 2 queens. The socio-genetic origin of queens correlated with their mode of colony-founding (Table 1). Only 5 out the 56 incipient nests established by monogyne queens had multiple queens (2 or 3 queens), whereas 1 out of the 2 colonies established by polygyne queens was multi-queened, with the greatest number of queens (5). All nests with multiple queens had either only monogyne or only polygyne queens. Colonies with a single queen were smaller than colonies with multiple queens (model 2, estimate ± SE =  − 1.33 ± 0.51, *p* = 0.0085; Online Resource Fig. S2 and Table S2).

All but one queen were inseminated, and queens had mated mostly assortatively with respect to social form (model 3, intercept estimate ± SE = 1.15 ± 0.31, *p* < 0.001; socio-genetic origin of queens, *p* = 0.84; Table 1 and Online Resource Table S3). Specifically, 75.9% of the monogyne queens had mated to *M* male(s), and 80% of the polygyne queens had mated to *P* male(s).

### Independent colony-founding in the laboratory

#### Are polygyne queens more prone to start colonies cooperatively?

**Fig. 1:**
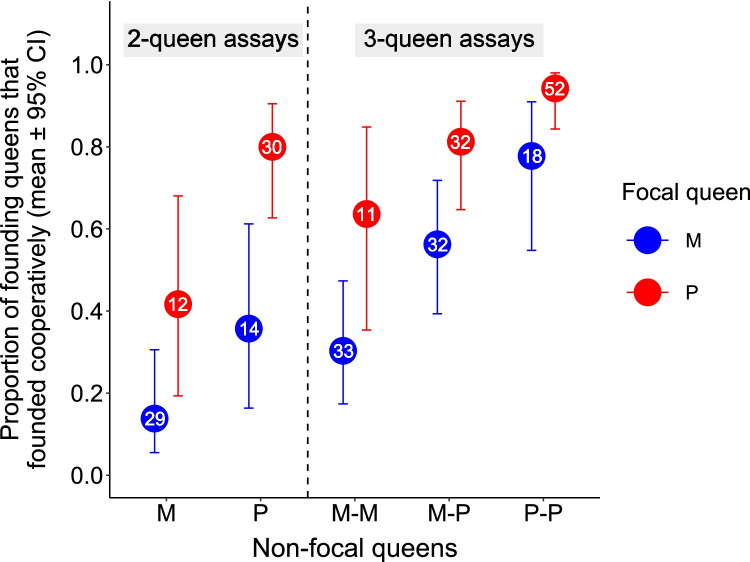
Propensity of monogyne (M, blue) and polygyne (P, red) queens to found colonies cooperatively in two-queen (left) and three-queen (right) assays, according to the social origin of the other queen(s) in the assay (non-focal queens). All queens included in this plot founded a colony. Each non-focal queen is represented by one letter. The number of focal queens is displayed inside circles

Monogyne and polygyne queens in the laboratory were as likely to survive during the first week (model 9, *p* = 0.99; Online Resource Fig. S3 and Table S4) and were as likely to start a nest independently (model 6, *p* = 0.97; Online Resource Fig. S4 and Table S5), but polygyne queens were more likely to found cooperatively than monogyne queens (Fig. 1 and Online Resource Fig. S5). In both two- and three-queen assays, the rates of colony-founding did not co-vary with the number of polygyne queens within assays (models 4, both *p* > 0.72; Online Resource Fig. S5 and Table S6), but the rates of cooperative colony-founding were higher in assays with a majority of polygyne queens (models 5, two-queen essays, *p* = 0.0013; post hoc comparisons, both *p*’ < 0.024; three-queen assays, *p* < 0.001; post hoc comparisons, all *p*’ < 0.047; Online Resource Fig. S5 and Table S7). Accordingly, polygyne queens were significantly more likely to found colonies cooperatively than monogyne queens (model 7, estimate ± SE = 6.30 ± 1.44, *p* < 0.0001; Fig. 1 and Online Resource Table S8), and both types of queens were significantly more likely to do so with polygyne than with monogyne queens (model 7, interaction, *p* = 0.11; main effect, *p* < 0.0001; post hoc comparison, estimate ± SE = 8.51 ± 1.70, *p*’ < 0.001; Fig. 1 and Online Resource Table S9).

At the onset of the experiment, polygyne queens were more likely to gather with other queens (model 8, estimate ± SE = 0.54 ± 0.22, *p* = 0.014; Online Resource Fig. S6 and Table S9), especially with polygyne queens (model 8, interaction, *p* = 0.009; post hoc comparison, estimate ± SE = 2.54 ± 0.88, *p*’ = 0.021; Online Resource Fig. S5 and Table S9), whereas monogyne queens had similar probabilities of gathering with monogyne and polygyne queens (model 8, post hoc comparison, estimate ± SE = 1.27 ± 0.81, *p*’ = 0.22; Online Resource Fig. S6 and Table S9). In addition, both monogyne and polygyne queens were more likely to fail at starting a nest and to die during the first week in the presence of monogyne than of polygyne queens (models 6 and 9, interaction, both *p* > 0.27; founding probability, *p* = 0.017; post hoc comparison, estimate ± SE = 0.66 ± 0.25, *p*’ = 0.026; death probability, *p* = 0.0016; post hoc comparison, estimate ± SE = 0.74 ± 0.22, *p*’ = 0.003; Online Resource Fig. S4 and Table S5 and Fig. S3 and Table S4, respectively). The number of queens in the assay had no effect on the propensity of queens to gather (model 8, estimate ± SE = 0.08 ± 0.30, *p* = 0.79; Online Resource Fig. S6 and Table S9), on the probability of queens to start a nest (model 6, estimate ± SE =  − 0.05 ± 0.25, *p* = 0.84; Online Resource Fig. S4 and Table S5), or on their likelihood to do it cooperatively (model 7, estimate ± SE = 2.81 ± 1.85, *p* = 0.13; Fig. 1 and Online Resource Table S8). The proportion of queens that died during the first week was higher in three-queen than in two-queen assays (model 9, estimate ± SE = 0.49 ± 0.21, *p* = 0.023; Online Resource Fig. S3 and Table S4).

#### Is cooperative colony-founding beneficial to queens?

**Fig. 2:**
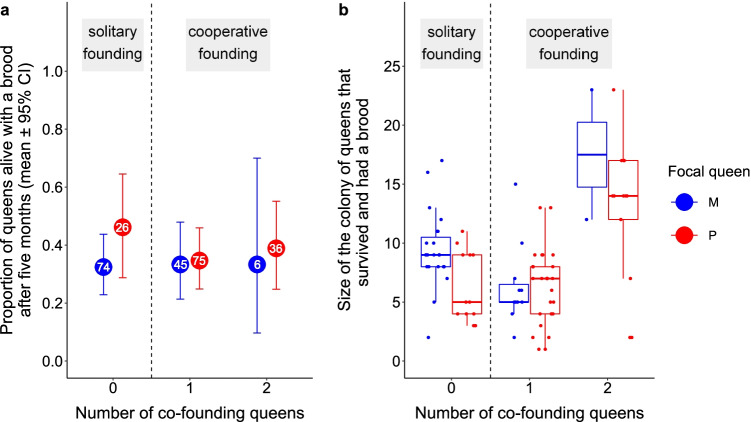
Colony-founding success of monogyne (M, blue) and polygyne (P, red) queens that founded solitarily or cooperatively. Colony-founding success is shown as **a** the proportion of queens that were alive and had a brood after 5 months and **b** the size of their colonies after 5 months. Box plots represent the median and the 1st and 3rd quartiles, and each dot represents one alive queen. The number of focal queens in **a** is displayed inside circles

Queens that founded a colony cooperatively with one or two queens had similar chances of being alive with a brood at the end of the experiment than queens that founded alone (model 10, *p* = 0.84; Fig. 2a and Online Resource Table S10), but queens that co-founded with two queens had larger colonies (model 11, *p* < 0.0001; post hoc comparisons, estimate ± SE (3Q–1Q) = 0.55 ± 0.18, *p*’ = 0.005; estimate ± SE (3Q–2Q) = 0.77 ± 0.18, *p*’ < 0.001; Fig. 2b and Online Resource Table S11). The queens that founded a colony alone produced colonies of similar size than those that co-founded with one queen (model 11, post hoc comparison, estimate ± SE = 0.22 ± 0.13, *p*’ = 0.1; Fig. 2b and Online Resource Table S11).

Monogyne and polygyne queens were as likely to survive and have a brood after 5 months (model 10, estimate ± SE = 0.12 ± 0.34, *p* = 0.73; Fig. 2a and Online Resource Table S10), independently of the number of queens with whom they founded (model 10, interaction, *p* = 0.62), but monogyne queens had slightly larger colonies at the end of the experiment (model 11, interaction, *p* = 0.41; main effect, estimate ± SE = 0.23 ± 0.11, *p* = 0.041; Fig. 2b and Online Resource Table S11). Specifically, monogyne queens that founded alone had colonies about 1.5 times larger than polygyne queens that founded alone (model 11, post hoc comparison, estimate ± SE = 0.39 ± 0.13, *p*’ = 0.006; Fig. 2b and Online Resource Table S11), whereas both types of queens had colonies of similar sizes when they founded cooperatively (model 11, post hoc comparison (2Q), *p*’ = 0.72; we did not carry out post hoc comparison between queens that co-founded with two queens due to low sample size; Fig. 2b and Online Resource Table S11). The social origin of the male with whom the queen mated did not affect the number of workers that she had in her colony at the end of the experiment (model 11, estimate ± SE = 0.09 ± 0.11, *p* = 0.41; Fig. 2b and Online Resource Table S11).

#### Does cooperative colony-founding by polygyne queens lead to permanent multi-queened colonies?

Of the 74 colonies that were founded cooperatively, 53 (72%) still had one or more queens after 5 months, of which 19 (36%) remained multi-queened and 34 (64%) became single-queened (Table 2). The likelihood that colonies became single-queened did not co-vary with the presence of offspring (proportion of colonies with workers in colonies that became single-queened or remained multi-queened, 73.5% and 78.9%, respectively; model 13, *p* = 0.85; Table 2 and Online Resource Table S12). Instead, the likelihood that colonies became single-queened co-varied with the presence of monogyne queens in the colony, as queens were more likely to die when they co-founded with monogyne queen(s) than when they co-founded with polygyne queen(s) (model 12, estimate ± SE =  − 0.84 ± 0.42, *p* = 0.049; Online Resource Tables S13 and S14), independently of their own social origin (model 12, interaction, *p* = 0.26; main effect, estimate ± SE = 0.45 ± 0.40, *p* = 0.26; Online Resource Tables S13 and S14). Accordingly, colonies with monogyne queen(s) were more likely to become single-queened than to remain multi-queened (intercept estimate ± SE = 0.85 ± 0.40, *z* = 2.13, *p* = 0.033; Table 2), whereas both outcomes had similar probabilities for colonies hosting polygyne queens exclusively (intercept estimate ± SE = 0.26 ± 0.42, *z* = 0.62, *p* = 0.53; Table 2). In addition, colonies co-founded by polygyne queens exclusively were about five times more likely to have both a brood and all co-founding queens alive at the end of the experiment than colonies co-founded by monogyne queen(s) (25.0% vs 5.3%, respectively; model 14, estimate ± SE = 1.84 ± 0.83, *p* = 0.027; Online Resource Fig. S7 and Table S15). The number of queens within colonies did not affect the survival probabilities of queens (model 12, *p* = 0.51; Online Resource Tables S13 and S14), but colonies with three queens had higher probabilities to remain multi-queened than colonies with two queens (model 13, estimate ± SE = 2.45 ± 0.87, *p* = 0.005; Online Resource Table S12).

**Table 2:** Number of colonies that started cooperatively and remained multi-queened became single-queened or had no queen alive after 5 months, according to the social origin of the co-founding queens (mixed = monogyne and polygyne). Numbers in the parentheses indicate colonies that produced at least one worker

	Social origin of queens in co-founded colonies		
	Monogyne	Mixed	Polygyne
Multi-queened	1 (1)	8 (4)	10 (10)
Single-queened	6 (4)	15 (12)	13 (9)
Failed	5	3	13

## Discussion

Plasticity in social organization has long been considered ubiquitous in group-living animals (Stacey and Bock 1978; Koenig et al. 1992; Wcislo 1997; Heinze 2008; Gadau et al. 2009; Maher and Burger 2011; Schradin et al. 2012). In many ant species, major features of colony social organization, such as queen number, multiple mating, worker number, reproductive skew, and aggressiveness change during colony development and/or according to certain ecological variables (Heinze 2008; Trettin et al. 2014). The recent findings that colony queen number is controlled by supergenes in multiple ant clades (Wang et al. 2013; Purcell et al. 2014; Braim 2015; Brelsford et al. 2020; Yan et al. 2020) have shaken the widespread assumption that this important component of ant social organization is plastic. They also raise novel questions about the behavioral mechanisms linking supergene variants to alternative forms of colony social organization.

Here, we show that *F. selysi* ant queens belonging to alternative social forms differ in their colony-founding behavior, both in the wild and in the laboratory. Queens originating from monogyne and polygyne colonies established incipient colonies independently, without worker assistance, but polygyne queens were more likely to do so cooperatively than monogyne queens. These associations yielded larger colonies, and they did not break up after the emergence of the first cohort of workers (Bernasconi and Strassmann 1999). Instead, they persisted for months, suggesting that cooperative colony-founding by polygyne queens can lead to permanent multi-queened colonies in nature. Our laboratory experiments further indicate that alternative supergene variants are associated with different levels of mutual attraction and social tolerance in young queens, making *P*-carrying queens cooperative during independent colony-founding. This hardwired behavioral mechanism contributes to perpetuate alternative forms of social organization.

Field sampling of incipient colonies showed that both monogyne and polygyne queens establish new colonies independently in the wild. This contrasts with the general view that only monogyny is associated with independent colony-founding in ants (e.g., Keller 1991; Keller 1993; Bourke and Franks 1995; Sundström et al. 2005; but see Hamidi et al. 2017). Several lines of evidence indicate that both types of queens mated in swarms and founded incipient colonies independently, without the help of workers. First, 24% of the monogyne and polygyne queens in incipient nests were mated to males of the alternative social form, indicating that they did not mate in their natal nest (Avril et al. 2019a, 2020). Second, the proportion of monogyne and polygyne queens was similar in incipient colonies and in natural mating aggregations (91% and 89.6% of monogyne queens, respectively; Fontcuberta et al. 2021), in line with the hypothesis that queens dispersed on the wing and mated in aggregations before to establish colonies. Last and foremost, all incipient colonies had fewer than 11 adult workers. This tiny colony size, and the fact that workers in incipient colonies were much smaller than workers in mature colonies, shows that queens founded incipient colonies independently, without being accompanied by adult workers from nearby colonies (Hölldobler and Wilson 1990; Fernández Escudero et al. 2001; Buczkowski and Bennett 2009; Cronin et al. 2012). Taken together, our results reveal that polygyne queens are not restricted to dependent colony-founding (Cronin et al. 2013), but that they also successfully engage in independent colony-founding in nature, which might be a common pattern in monodomous, polygyne species (Hamidi et al. 2017). Furthermore, field data also revealed that both solitary and cooperative colony-founding occur in the wild, which adds to the growing body of literature showing that cooperative colony-founding is a widespread mode of founding in ants (e.g.Bernasconi and Strassmann 1999; Johnson 2004; Shaffer et al. 2016; Gotoh et al. 2017; Madsen and Offenberg 2017; Masoni et al. 2017; Motro et al. 2017; Lenancker et al. 2019; Aron and Deneubourg 2020). Although monogyne and polygyne queens founded colonies independently, both solitarily and cooperatively, they differed markedly in their propensity to engage in cooperative colony-founding.

The socio-genetic origin of queens affected both the probability of cooperation during independent colony-founding and how long cooperation lasted. Polygyne queens were twice as likely as monogyne queens to associate with other queens when starting a nest. They were also more likely to form durable associations, as queens that associated with polygyne queens had lower chances of dying. The contrasting behavior of polygyne and monogyne queens helps explain how alternative social organizations emerge during colony development. Monogyne queens display low social tolerance towards other queens and typically form single-queen colonies. In contrast, polygyne queens exhibit high mutual attraction and social tolerance, which lead to multiple queening from the first stage of colony ontogeny. Cooperation of alien polygyne queens during independent colony-founding is also likely to explain the presence of unrelated queens within field polygyne colonies (Avril et al. 2019a; De Gasperin et al. 2021), as mature colonies do not adopt alien queens (Meunier et al. 2011; De Gasperin et al. 2021). Overall, cooperation between alien polygyne queens during independent colony-founding, together with queen re-adoption (Avril et al. 2019a) and potentially colony fusion (De Gasperin et al. 2021), is a hardwired proximate mechanism leading to polygyne social organization.

Interestingly, monogyne queens sometimes formed durable associations with other queens in the laboratory, suggesting that these queens may also form mature multiple-queen colonies through cooperative colony-founding in nature. Rare colonies hosting a couple of monogyne queens have indeed been described in the wild (Purcell et al. 2014), but so far associations of monogyne and polygyne queens have never been detected, be it in incipient or mature field colonies (Purcell et al. 2014; Avril et al. 2019a).

Why do alien queens associate to start new colonies? And why do polygyne queens have higher propensities to do so than monogyne queens? Associating with alien queens can be beneficial for each queen if it improves colony growth, as larger incipient colonies have better foraging abilities and better resistance to competitors, predators, or nest usurpers (Markin et al. 1973; Oster and Wilson 1978; Tschinkel and Howard 1983; Rissing and Pollock 1987; Jerome et al. 1998; Eriksson et al. 2019). Cooperative colony-founding did not affect the queens’ likelihood to survive and raise a brood, compared to solitary colony-founding. Instead, queen associations often resulted in larger colony size compared to solitary founding, in both the field and laboratory. This was especially true for polygyne queens, which were less fertile than monogyne queens in the solitary mode of founding (see also Reber et al. 2010; Avril et al. 2019b; De Gasperin et al. 2020). Therefore, cooperative colony-founding provided greater benefits to polygyne queens than to monogyne queens, in terms of colony growth. We suggest that polygyne queens associate during colony-founding to benefit from a rapid increase in colony workforce. As polygyne queens are smaller than monogyne queens (Meunier and Chapuisat 2009; Reber et al. 2010), cooperative colony defense might also contribute to their greater propensity to associate during colony-founding.

To conclude, we uncovered a link between the socio-genetic origin of queens and their propensity to cooperate and form long-lasting associations during independent colony-founding in a socially polymorphic ant species. Both monogyne and polygyne queens founded nest independently in the field and in the laboratory, but polygyne — *P*-carrying — queens had a higher propensity of doing so cooperatively with others *P*-carrying queens, as these queens attract each other and are socially tolerant. This difference in the level of cooperation during independent colony-founding between monogyne and polygyne queens is a proximate mechanism by which supergene variants produce alternative forms of social organization (Purcell et al. 2014; Avril et al. 2019a). Across social insects, cooperative colony-founding appears widespread and can help explain why unrelated reproductive queens co-inhabit colonies (Schwarz 1987; Stille and Stille 1992; Bourke and Franks 1995; Seppä 1996; Goodisman and Ross 1999; Queller et al. 2000; Zinck et al. 2007; Boomsma et al. 2014; Avril et al. 2019a; De Gasperin et al. 2021). Overall, this study provided new insights into the mechanisms leading to alternative social organizations in ants and opened new avenues for understanding how simple phenotypic differences at the individual level result in large differences at higher levels of organization.

## Supplementary Information

Below is the link to the electronic supplementary material.

Supplementary file1: Supplementary figures and tables (PDF 8177 KB)

Supplementary file2: Dataset of queens in the laboratory (XLSX 73 KB)

Supplementary file3: Dataset of queens in the field (XLSX 15 KB)

Supplementary file4: Dataset of workers' body size in the field (XLSX 20 KB)

Supplementary file5: Dataset of queen assays in the laboratory (XLSX 19 KB)

Supplementary file6: Dataset of co-founded colonies in the laboratory (XLSX 15 KB)

## Data Availability

The datasets generated and/or analyzed during the current study are available within the article and its supplementary materials.
