# Supplementary material for: Cooperation by ant queens during colony-founding perpetuates alternative forms of social organization
Source: Behav Ecol Sociobiol. 2021 Nov 30;75(12):165. doi: 10.1007/s00265-021-03105-1 (PMC8718384; doi:10.1007/s00265-021-03105-1)
Supplement: Supplementary file 1 — Supplementary file1: Supplementary figures and tables (PDF 8177 KB) [file 265_2021_3105_MOESM1_ESM.pdf]

Cooperation by ant queens during colony-founding perpetuates alternative forms of social organization

Pierre Blacher, Ornela De Gasperin, Michel Chapuisat

Department of Ecology and Evolution,

University of Lausanne,

Lausanne, Switzerland

Correspondence:

Pierre Blacher

E-mail: pierre.blacher@unil.ch

ORCID:

Pierre Blacher: 0000-0002-4255-4277

Ornela De Gasperin: 0000-0003-3415-2072

Michel Chapuisat: 0000-0001-7207-199X

Supplementary Information

Figures

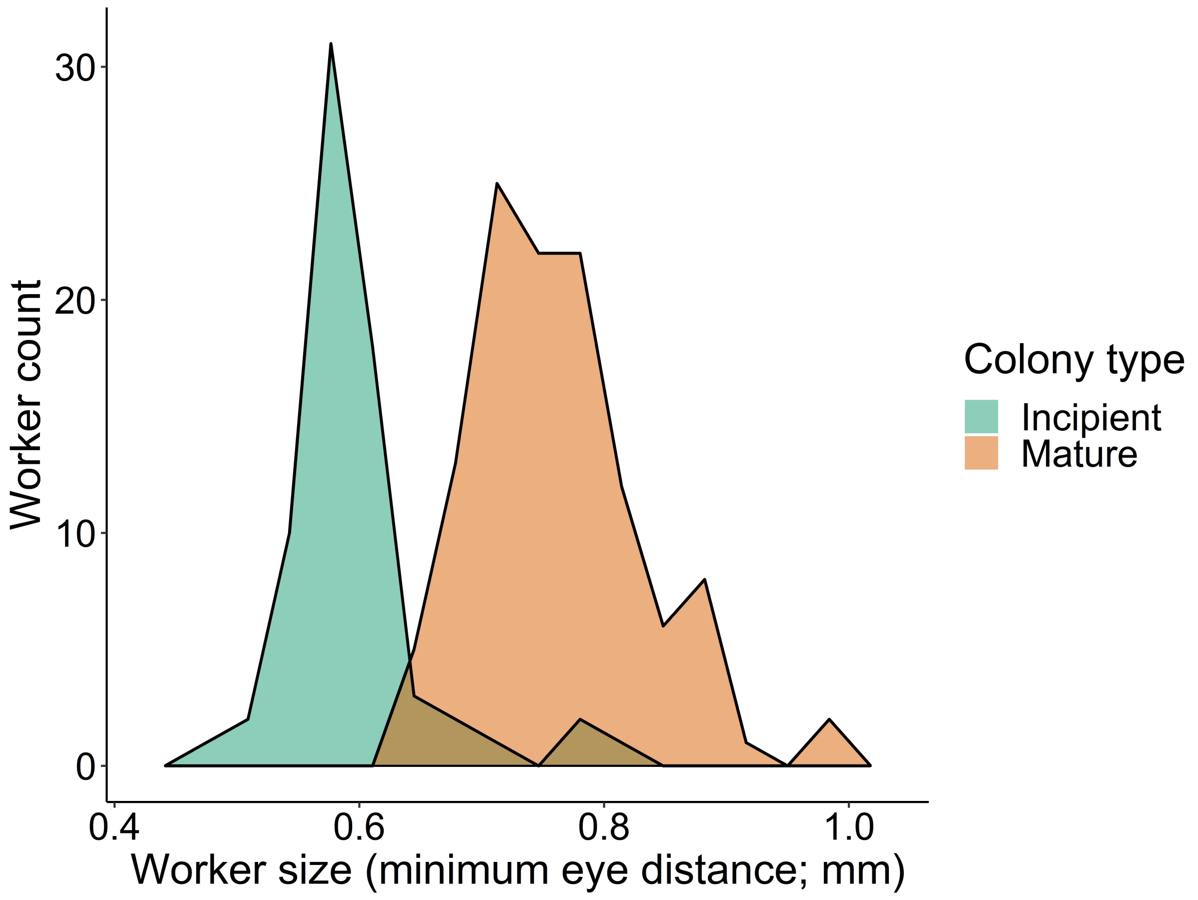

Fig. S1 Size distribution of workers in incipient (green) and mature (orange) field colonies

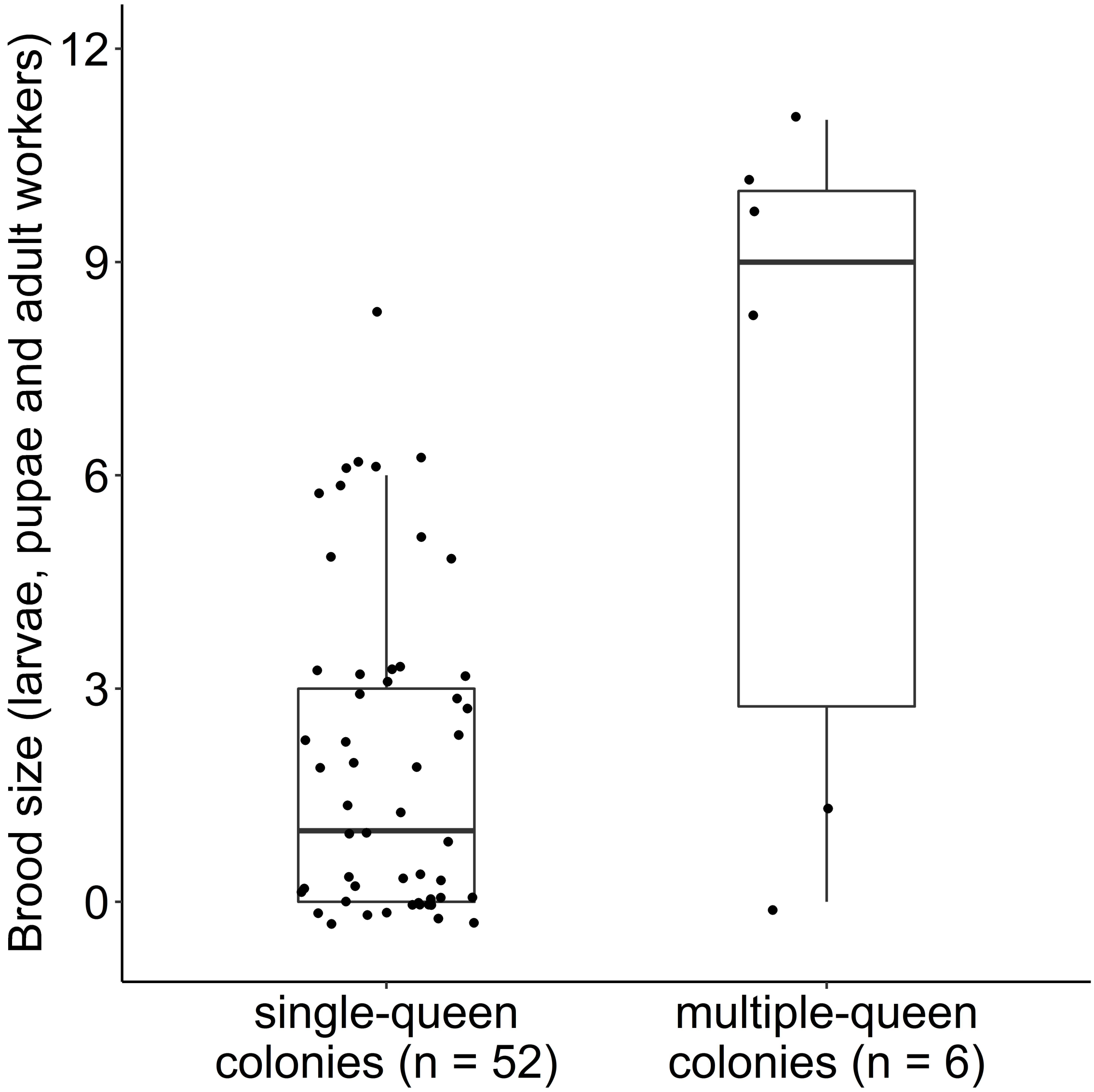

Fig. S2 Brood size of incipient single-queen and multiple-queen field colonies. Box plots represent the median, the 1st and 3rd quartiles, and the non-outlier range. Each dot represents a colony

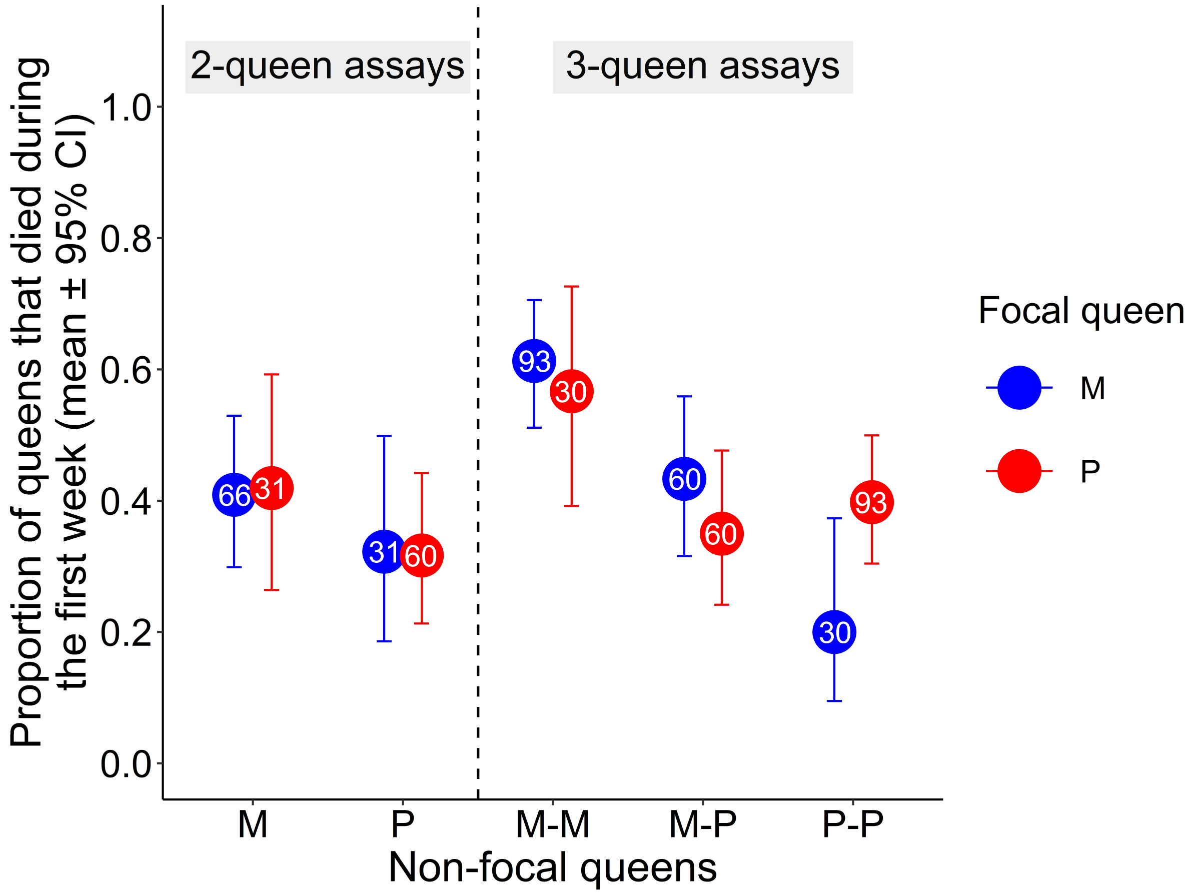

Fig. S3 Mortality of monogyne (M, blue) and polygyne (P, red) queens after one week in two-queen (left) and three-queen (right) assays, according to the social origin of the other queen(s) in the assay (non-focal queens). Each non-focal queen is represented by one letter. Number of focal queens is displayed inside circles

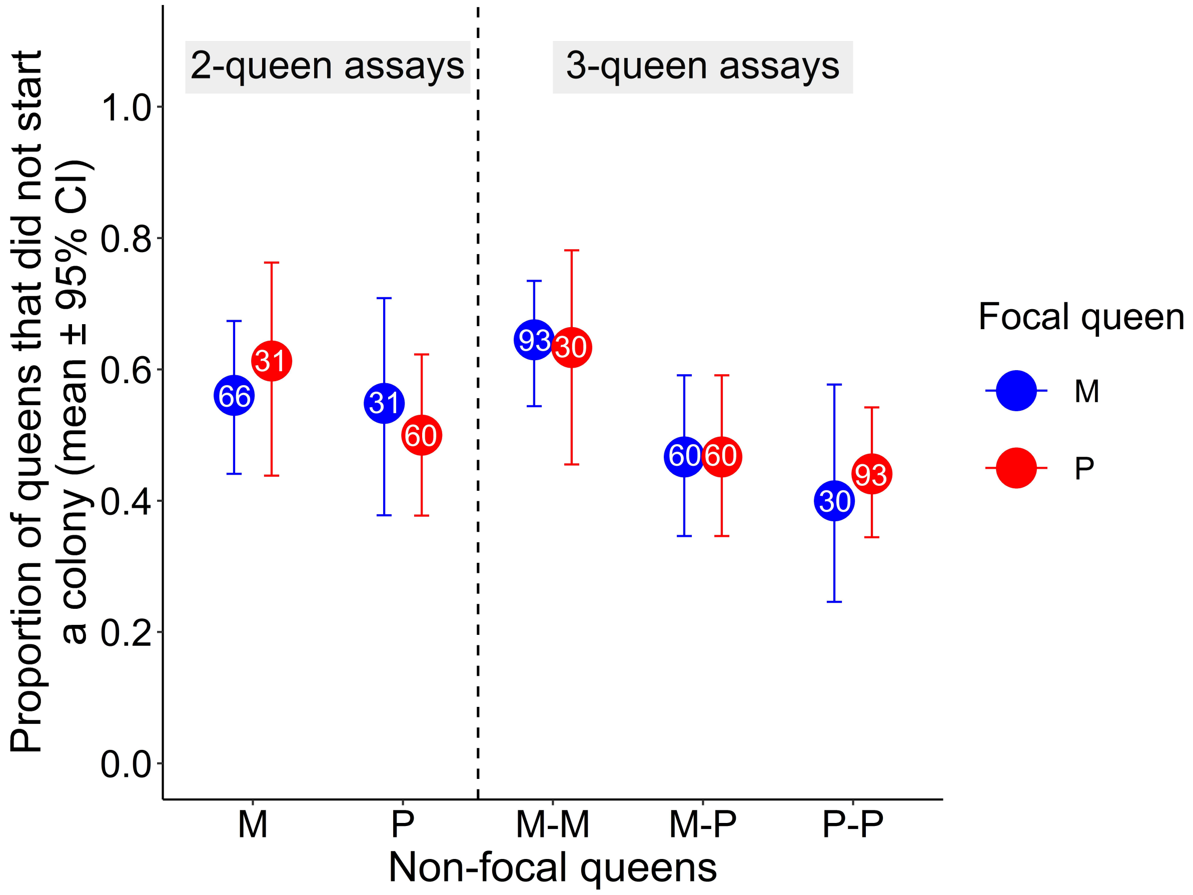

Fig. S4 Failure of monogyne (M, blue) and polygyne (P, red) queens at starting a colony in two-queen (left) and three-queen (right) assays, according to the social origin of the other queen(s) in the assay (non-focal queens). Each non-focal queen is represented by one letter. Queens failed at starting a nest when they remained on sand surface throughout the experiment (or until their death). Number of focal queens is displayed inside circles

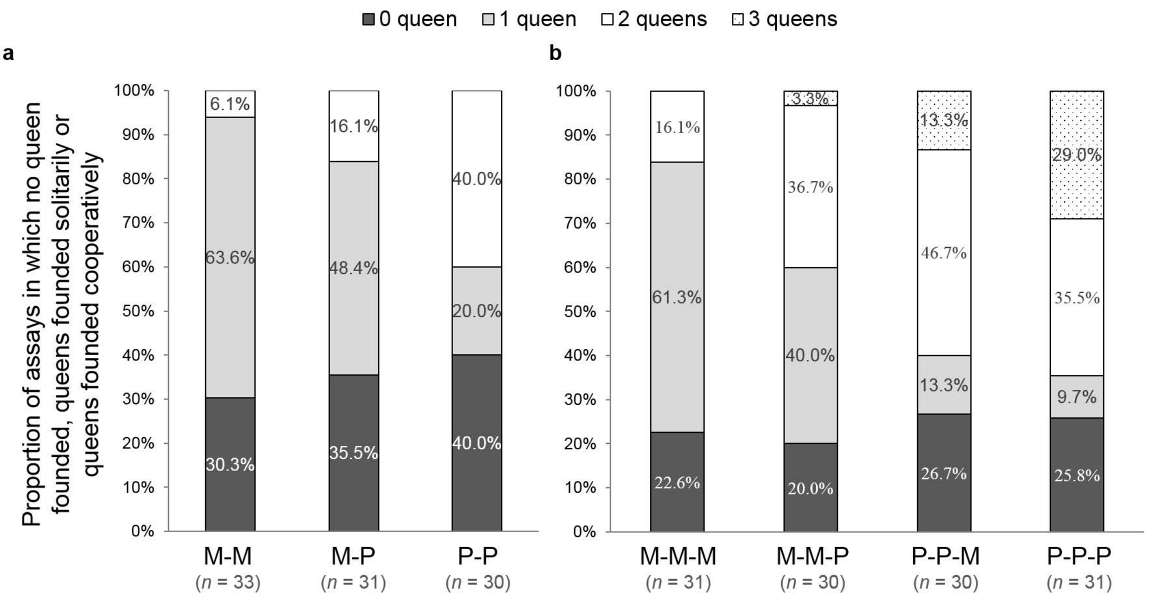

Fig. S5 Colony founding behavior exhibited by monogyne (M) and polygyne (P) queen in (a) two-queen and (b) three-queen assays. The social origin of the queens in the assays is indicated below the bars, with each letter representing one queen. Colony founding was characterized by observations of queens inside excavated sand cavities. Bars indicate the proportions of assays in which none of the queens founded a nest (black bars), at least one queen founded solitarily (grey bars) or queens founded cooperatively in association of two (white bar) or three queens (white bars with black dots). In 3 assays (2 P-P-M assays and 1 M-M-P assay), both solitary (1 queen) and cooperative (2 queens) colony-founding occurred. The outcome of these assays was categorized as 2-queen association. n refers to the number of assays

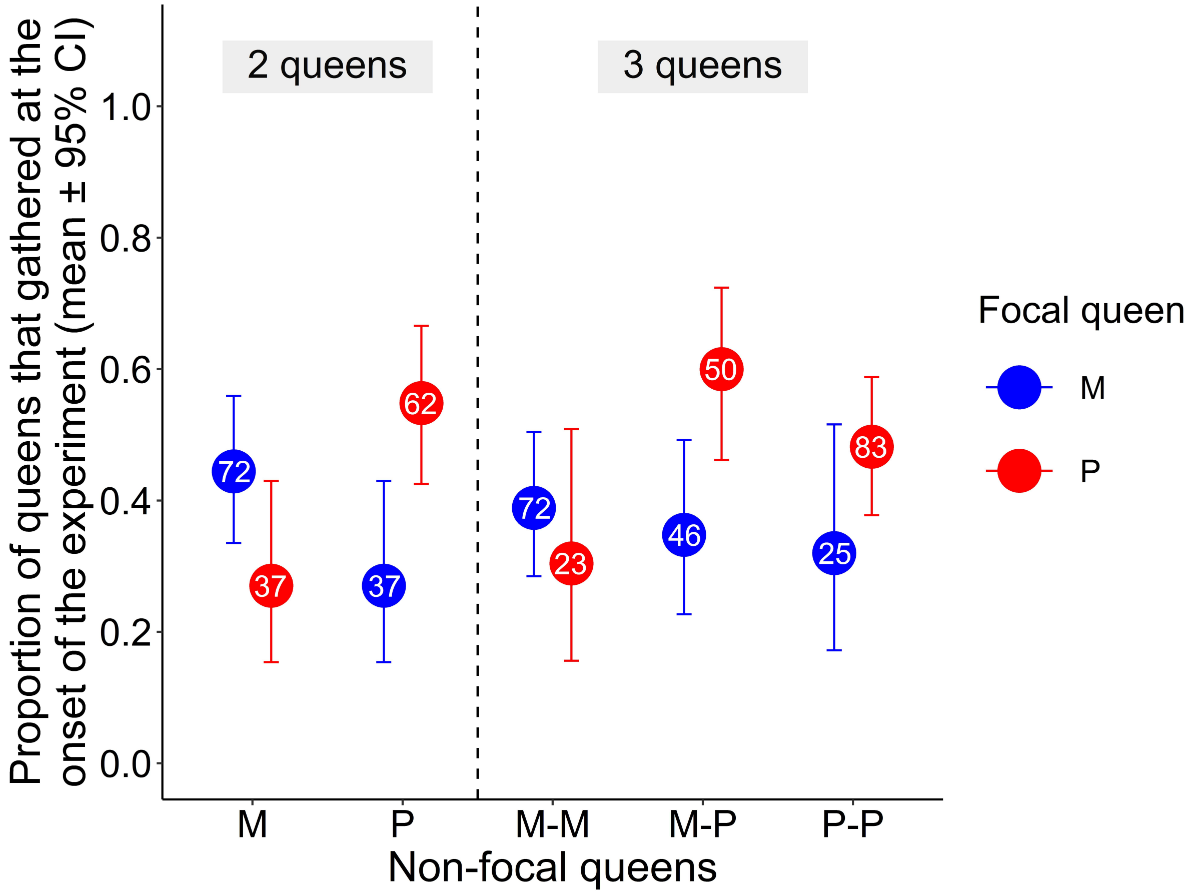

Fig. S6 Propensity of monogyne (M, blue) and polygyne (P, red) queens to gather in assays with two (left) and three (right) queens, according to the social origin of the other queen(s) in the assay (non-focal queens). Each non-focal queen is represented by one letter. Queens gathered when they were spatially close to another queen during the first observation. Number of focal queens is displayed inside circles

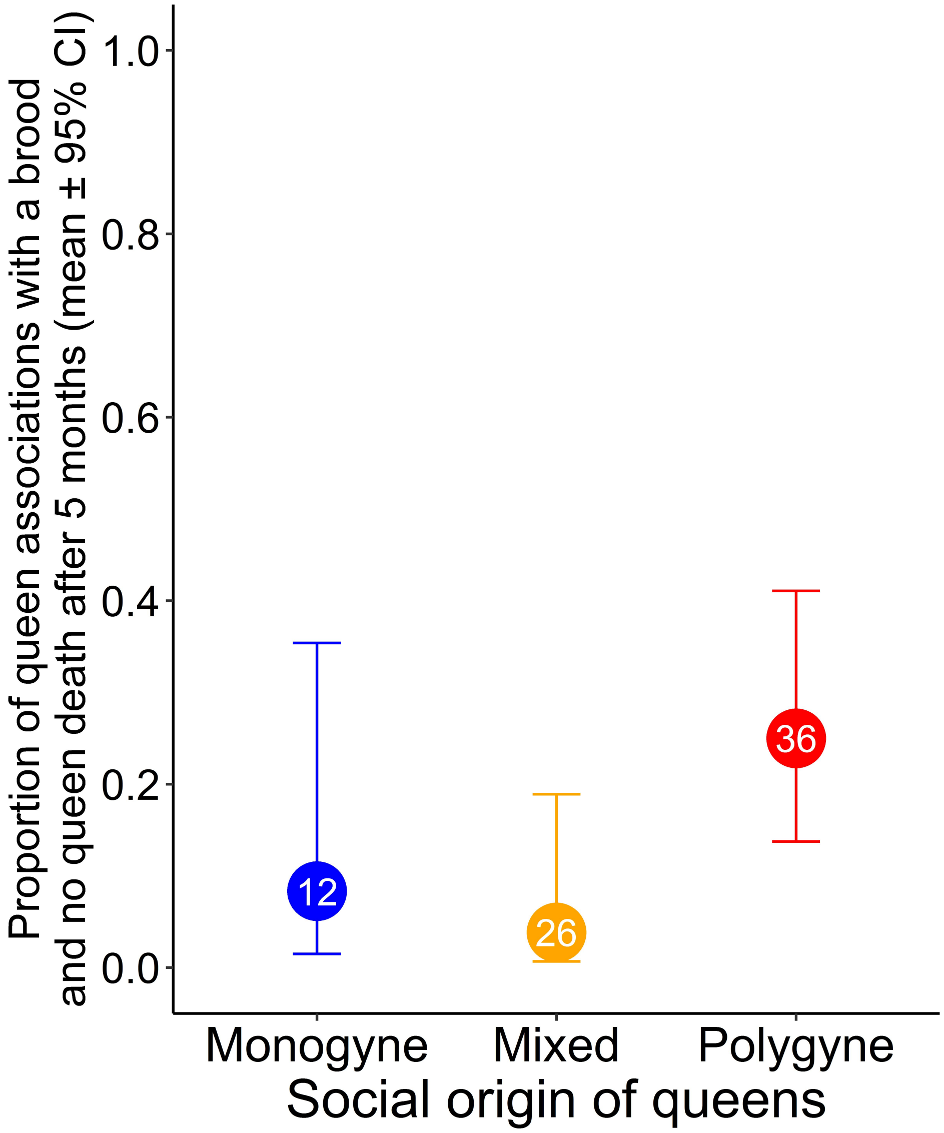

Fig. S7 Stability of queen associations according to the social origin of queens. Blue represents associations of monogyne queens only, orange of both monogyne and polygyne queens and red of polygyne queens. Number of queen associations is displayed inside circles

Tables

Table S1. Model 1: GLMM fitted to test whether the body size of workers differed between incipient and mature colonies. The colony of origin of the workers was included as a random factor.

Factor	Comparison	*p*	Estimate	SE	*z* score
Type of colony	Incipient – Mature	*< 0.0001*	-0.16	0.01	-11.06

Table S2. Model 2: GLMM fitted to test whether colony size differed between single-queen (SQ) and multiple-queen (MQ) incipient field colonies. An observation level random effect (olre) was included to account for overdispersion.

Factor	Comparison	*p*	Estimate	SE	*z* score
Type of colony	SQ – MQ	*0.0085*	-1.33	0.51	-2.63

Table S3. Model 3: GLMM fitted to test whether queens and males had mated assortatively with respect to social form. The colony of origin of the queens was included as a random factor.

Factor	Comparison	*p*	Estimate	SE	*z* score
Intercept		*< 0.001*	1.15	0.31	3.73
Queen social form	M – P	*0.84*	-0.24	1.16	-0.21

Table S4. Model 9: GLMM fitted to test the mortality of queens after one week. The colony of origin of the queens and the assay id nested within the year were included as random factors. The model includes an interaction term for the two first factors. Estimates for main factors were calculated after removing the non-significant interaction term. Post hoc tests were corrected for multiple comparisons using the Benjamini-Hochberg procedure (FDR method).

Factor	Comparison	p (χ^2, df)	Estimate	SE	*z* score
Social origin focal	M – P	*0.99*	0.01	0.23	0.01
Social origin non-focal	overall	*0.0016* (12.8, *2*)			
	M – Mix	*0.011*	0.71	0.26	2.70
	M – P	*0.0031*	0.74	0.22	3.30
	Mix – P	*0.90*	0.03	0.26	0.13
Interaction	overall	*0.27* (2.65, *2*)			
Type of assay	2Q – 3Q	*0.023*	-0.49	0.21	-2.27

Table S5. Model 6: GLMM fitted to test the probability that queens failed at starting a colony. The colony of origin of the queens and the assay id nested within the year were included as random factors. The model includes an interaction term for the two first factors. Estimates for main factors were calculated after removing the non-significant interaction term. Post hoc tests were corrected for multiple comparisons using the Benjamini-Hochberg procedure (FDR method).

Factor	Comparison	p (χ^2, df)	Estimate	SE	z score
Social origin focal	M – P	*0.97*	0.01	0.24	0.04
Social origin non-focal	overall	*0.017* (8.16, *2*)			
	M – Mix	*0.049*	0.64	0.30	2.14
	M – P	*0.026*	0.66	0.25	2.63
	Mix – P	*0.93*	0.02	0.29	0.08
Interaction	overall	*0.99* (0.02, *2*)			
Type of assay	2Q – 3Q	*0.84*	0.05	0.25	0.20

Table S6. Models 4: GLMs fitted to compare between treatments the probability that all queens within assays failed at starting a colony.

Factor	Comparison	p (χ^2, df)
Treatment (2-queen assays)	overall	*0.72* (0.65, *2*)
Treatment (3-queen assays)	overall	*0.93* (0.47, *3*)

Table S7. Models 5: GLMs fitted to compare between treatments the probability that queens started their colonies solitarily or cooperatively. Post hoc tests were corrected for multiple comparisons using the Benjamini-Hochberg procedure (FDR method).

Factor	Comparison	p (χ^2, df)	Estimate	SE	z score
Treatment (2-queen assays)	overall	*0.001* (13.22, *2*)			
	M-M – M-P	*0.17*	-1.25	0.90	-1.39
	M-M – P-P	*0.0036*	-3.04	0.89	-3.41
	M-P – P-P	*0.023*	-1.79	0.72	-2.49
Treatment (3-queen assays)	overall	< *0.001* (22.5, *3*)			
	M-M-M – M-M-P	*0.051*	-1.33	0.65	-2.06
	M-M-M – P-P-M	*0.0008*	-2.84	0.75	-3.80
	M-M-M – P-P-P	*0.0006*	-3.23	0.80	-4.05
	M-M-P – P-P-M	*0.047*	-1.5	0.69	-2.19
	M-M-P – P-P-P	*0.024*	-1.90	0.74	-2.56
	P-P-M – P-P-P	*0.64*	-0.39	0.83	-0.47

Table S8. Model 7: GLMM fitted to test the probability that queens started their colonies solitarily or cooperatively. The colony of origin of the queens and the assay id nested within the year were included as random factors. Because the initial model encountered quasi-complete separation (see statistic section in the main text), the model was fit with a weak prior on the fixed effects. The model includes an interaction term for the two first factors. Estimates for main factors were calculated after removing the non-significant interaction term. Post hoc tests were corrected for multiple comparisons using the Benjamini-Hochberg procedure (FDR method).

Factor	Comparison	p (χ^2, df)	Estimate	SE	z score
Social origin focal	M – P	< *0.001*	-6.30	1.44	-4.36
Social origin non-focal	overall	< *0.001* (25.2, *2*)			
	M – Mix	*0.006*	-4.13	1.50	-2.76
	M – P	< *0.001*	-8.51	1.70	-5.02
	Mix – P	*0.006*	-4.37	1.57	-2.78
Interaction		*0.11* (4.43, *2*)			
Type of assay	2Q – 3Q	*0.13*	-2.81	1.85	-1.52

Table S9. Model 8: GLMM fitted to test the probability that queens gathered at the onset of the experiment. The colony of origin of the queens and the assay id nested within the year were included as random factors. The model includes an interaction term for the two first factors. Estimates for main factors were calculated with type III sums of squares. Post hoc tests were corrected for multiple comparisons using the Benjamini-Hochberg procedure (FDR method).

Factor	Comparison	p (χ^2, df)	Estimate	SE	z score
Social origin focal	M – P	*0.014*	-0.54	0.22	-2.46
Social origin non-focal	overall	*0.25* (2.79, *2*)			
Interaction	overall	*0.009* (9.5, *2*)			
	M-M – P-M	*0.20*	1.40	0.83	1.69
	M-M – M-Mix	*0.45*	0.92	0.94	0.98
	M-M – P-Mix	*0.23*	-1.31	0.91	-1.43
	M-M – M-P	*0.22*	1.27	0.81	1.56
	M-M – P-P	*0.23*	-1.14	0.79	-1.45
	P-M – M-Mix	*0.63*	-0.48	0.72	-0.67
	P-M – P-Mix	*0.019*	-2.71	0.89	-3.04
	P-M – M-P	*0.85*	-0.13	0.68	-0.20
	P-M – P-P	*0.021*	-2.54	0.88	-2.88
	M-Mix – P-Mix	*0.081*	-2.23	1.01	-2.22
	M-Mix – M-P	*0.80*	0.34	0.87	0.40
	M-Mix – P-P	*0.09*	-2.06	0.99	-2.09
	P-Mix – M-P	*0.01*	2.58	0.75	3.41
	P-Mix – P-P	*0.85*	0.17	0.91	0.18
	M-P – P-P	*0.023*	-2.41	0.87	-2.76
Queen number	2Q – 3Q	*0.79*	0.08	0.30	0.26

Table S10. Model 10: GLMM fitted to test the probability that queens were alive with a brood at the end of the experiment. The colony of origin of the queens and the nest id nested within the assay id nested within the year were included as random factors. The model includes an interaction term for the two first factors. Estimates for main factors were calculated after removing the non-significant interaction term. Post hoc tests were corrected for multiple comparisons using the Benjamini-Hochberg procedure (FDR method).

Factor	Comparison	*p* (χ^2, *df*)	Estimate	SE	*z* score
Social origin focal	M – P	*0.73*	-0.12	0.34	-0.35
Number of co-founding queens	overall	*0.84* (0.35, *2*)			
Interaction	overall	*0.62* (0.97, *2*)			
	M – P (1Q)	*0.97*	-0.39	0.56	-0.70
	M – P (2Q)	*0.97*	-0.02	0.45	-0.04
	M – P (3Q)	*0.97*	-0.23	1.29	-0.18

Table S11. Model 11: GLMM fitted to test the colony size of the queens that survived and had a brood after five months. The colony of origin of the queens and the nest id nested within the assay id nested within the year were included as random factors. The model includes an interaction term for the two first factors. Estimates for main factors were calculated after removing the non-significant interaction term. The M – P (3Q) comparison was omitted due to low sample size. Post hoc tests were corrected for multiple comparisons using the Benjamini-Hochberg procedure (FDR method).

Factor	Comparison	*p* (χ^2, *df*)	Estimate	SE	*z* score
Social origin focal	M – P	*0.041*	0.23	0.11	2.05
Number of founding queens	overall	< *0.001* (18.6, *2*)			
	1Q – 2Q	*0.1*	0.22	0.13	1.69
	1Q – 3Q	*0.005*	-0.55	0.18	-3.02
	2Q – 3Q	*0.0001*	-0.77	0.18	-4.30
Interaction	overall	*0.41* (1.78, *2*)			
	M – P (1Q)	*0.006*	0.39	0.13	2.95
	M – P (2Q)	*0.72*	0.08	0.21	0.36
Social origin mate	M – P	*0.41*	-0.09	0.11	-0.82

Table S12. Model 13: GLM fitted to test the probability that colonies started cooperatively became singly-queened or remained multi-queened after 5 months.

Factor	Comparison	*p*	Estimate	SE	*z* score
Social origin queens	(M/MP) – P	*0.39*	0.56	0.65	0.86
Brood	No – Yes	*0.85*	0.15	0.76	0.19
Number of co-founding queens	2Q – 3Q	*0.005*	2.45	0.87	2.81

Table S13. Survival probability after five months of monogyne (M) and polygyne (P) queens (focal queens) according to their mode of founding and to the social origin of the queens that co-founded with them (non-focal queens). Number of focal queens is indicated in parentheses.

	Non-founding		Solitary founding		Cooperative founding					
Focal queens	M (153)	P (137)	M (74)	P (26)	M			P		
Co-founding queens	-	-	-	-	M (24)	mixed (2)	P (25)	M (22)	mixed (8)	P (81)
Survival	0.07	0.07	0.41	0.54	0.33	1	0.64	0.36	0.62	0.43

Table S14. Model 12: GLMM fitted to test the probability that co-founding queens were dead at the end of the experiment. The colony of origin of the queens and the nest id nested within the assay id nested within the year were included as random factors. The model includes an interaction term for the two first factors. Estimates for main factors were calculated after removing the non-significant interaction term.

Factor	Comparison	*p* (χ^2, *df*)	Estimate	SE	*z* score
Social origin focal	M – P	*0.26*	-0.45	0.40	-1.12
Social origin non-focal	M – P	*0.049*	0.84	0.42	1.97
Interaction	overall	*0.26* (1.29, *1*)			
Number of co-founding queens	2Q – 3Q	*0.51*	-0.31	0.48	-0.65

Table S15. Model 14: GLM fitted to test whether co-founded colonies with and without monogyne queens differed in their likelihood to have a brood and all their co-founding queens alive at the end of the experiment.

Factor	Comparison	*p* (χ^2, *df*)	Estimate	SE	*z* score
Social origin queens	(M/MP) – P	*0.027*	-1.84	0.83	-2.21
Number of co-founding queens	2Q – 3Q	*0.67*	0.37	0.88	0.42
